# Unconventional Room‐Temperature Antisymmetric Magnetoresistance in van der Waals Fe_3_GaTe_2_/Pt Heterostructures

**DOI:** 10.1002/advs.76053

**Published:** 2026-06-09

**Authors:** Yunwen Zhu, Xiaolin Luo, Fan Gong, Jinnan Liu, Zhuang Liu, Jianlei Shen, Jinjian Guo, Baijie Zhu, Wei Zhang, Zhiyong Quan, Xiaohong Xu

**Affiliations:** ^1^ School of Materials Science and Engineering Key Laboratory of Magnetic Molecules and Magnetic Information Materials of Ministry of Education Shanxi Normal University Taiyuan China; ^2^ Research Institute of Materials Science Shanxi Key Laboratory of Advanced Magnetic Materials and Devices Shanxi Normal University Taiyuan China; ^3^ Instrumental Analysis Center Shanxi Normal University Taiyuan China

**Keywords:** room‐temperature antisymmetric magnetoresistance, spin‐momentum locking, van der Waals ferromagnetic heterostructures

## Abstract

Magnetoresistance in the 2D ferromagnetic van der Waals (vdW) Fe_3_GaTe_2_ (FGT) has emerged as a new frontier in spintronics, with particular interest paid to the antisymmetric magnetoresistance (AsMR) effect due to its potential to realize multi‐state memory, promising for constructing energy‐efficient memory devices. However, the mechanism underlying the room‐temperature AsMR remains unresolved. Herein, a structural design of two completely isolated FGT nanoflakes, combined with measurement approaches using swapping electrodes and flipping device orientations, was used to clarify the physics of room‐temperature AsMR in vdW ferromagnetic FGT‐based systems. The results show the unambiguous presence of room‐temperature AsMR with four distinct resistance states in the FGT/Pt Hall bar devices. Spin‐momentum locking is identified in the vdW FGT‐based heterostructures and found to be responsible for the observed room‐temperature AsMR. The special design and magneto‐electric transport measurements rule out the magnetic domain wall–induced circulating currents and interface pinning as possible origins of AsMR. Further confirmation of this physical mechanism is provided by the distinctive configuration of two FGT nanoflakes separated by a micrometer‐scale gap. Overall, the physical mechanism of room‐temperature AsMR in vdW FGT/Pt heterostructures is experimentally confirmed, opening new avenues for low‐power room‐temperature spintronic devices.

## Graphical abstract

We investigated a special electrical phenomenon, known as antisymmetric magnetoresistance with four distinct resistance states, in 2D Fe_3_GaTe_2_/Pt devices at room temperature. By designing devices with two separated magnetic nanoflakes, we confirmed that the effect originates from spin–momentum locking rather than magnetic domain walls or interface pinning. This finding opens new avenues for low‐power, multi‐state spintronic memory devices.

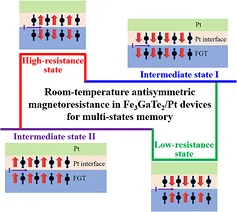

## 1 Introduction

The rapid development of new‐generation information technologies, such as artificial intelligence and the Internet of Things, requires high‐performance information storage and logic devices. Magnetoresistance effects, such as giant magnetoresistance and tunneling magnetoresistance, are important physical phenomena for memory and logic technologies [1, 2, 3, 4, 5]. An antisymmetric magnetoresistance (AsMR) effect, characterized by a non‐symmetric response of resistance to the direction of the magnetic field, has been discovered in conventional rare earth/transition metal alloy systems (such as CoTb and SmCo) and multilayer film systems (such as Co/Pt and Ni/Pt) [6, 7, 8]. Recently, 2D ferromagnetic van der Waals (vdW) materials (such as Fe_5_GeTe_2_ and Fe_5‐x_GeTe_2_), characterized by ultraclean interfaces and intrinsic strong ferromagnetism, have attracted considerable attention, especially in terms of their manifested intriguing AsMR behavior [9, 10, 11, 12, 13, 14]. Particular interest has also been paid to the 2D ferromagnetic vdW Fe_3_GaTe_2_ (FGT) with its high Curie temperature (*T*_C_, ∼350–380 K) and large perpendicular magnetic anisotropy (PMA) [15, 16], as well as the occurrence of the AsMR effect at room temperature [17, 18].

Advanced applications of room‐temperature 2D spintronic devices require clarification of the physical mechanism underlying room‐temperature AsMR in vdW ferromagnetic FGT‐based systems. Among these, Hu et al. observed room‐temperature AsMR in FGT nanosheets that emerged from a circulating current near the boundaries of domain walls [17]. Gao et al. attributed the room‐temperature AsMR behavior, induced by the folding of a continuous flat FGT nanosheet, to the circulating current in the vicinity of the domain walls [18]. With respect to 2D ferromagnetic vdW materials, the emergence of AsMR can primarily be attributed to three possible scenarios: (i) Domain wall circulating current effect [11, 12, 13, 17, 18]: In magnetic films with perpendicular magnetic anisotropy, magnetic domains separated by domain walls can form during the magnetization reversal process. When an electrical current passes across the domain walls, the anomalous Hall effect induces circulating currents in the vicinity of the domain walls, thereby contributing to the longitudinal resistance. Consequently, distinct resistance states may emerge during magnetic field sweeps, giving rise to the AsMR signal. (ii) Interfacial pinning effect [10]: In magnetic heterostructures, unsynchronized magnetization switching may occur due to interfacial exchange coupling. Under an external magnetic field, the magnetization can rotate more easily along certain directions, whereas it becomes pinned along others because of the interfacial pinning effect. This asymmetric magnetization reversal process can lead to different resistance states during field sweeps. (iii) Spin‐momentum locking effect [9, 14, 19]: In heterostructures with broken structural inversion symmetry and strong spin‐orbit coupling (SOC), spin‐momentum locking can generate spin‐polarized currents at the interface. Depending on the current direction, the spin polarization may become either parallel or antiparallel to the magnetization of the ferromagnetic layer, leading to distinct resistance states and consequently giving rise to a pronounced AsMR effect. Among these, the domain wall circulating current and spin‐momentum locking effect cannot easily be disentangled, as they exhibit similar experimental signatures with respect to sweeping field and electrode position measurements [9, 14, 17, 18]. Therefore, more direct experimental evidence and careful design are required to unambiguously elucidate the physical origin of room‐temperature AsMR in 2D vdW FGT‐based systems.

Herein, a structural design of two completely isolated FGT nanoflakes was used to clarify the physical mechanism of the room‐temperature AsMR in FGT‐based devices. This special design can intrinsically suppress the formation of magnetic domain walls between FGT nanosheets with different thicknesses, thereby ruling out the domain‐wall‐induced circulating current scenario as a plausible origin of the room‐temperature AsMR. The results show an unconventional AsMR, manifesting as four distinct states under an applied magnetic field in FGT/Pt Hall bar devices. This AsMR behavior originates from the spin‐momentum locking‐induced spin polarization at the FGT/Pt interface, confirmed by sweeping electrode measurements. Further verification of this mechanism is provided by the distinctive configuration consisting of two FGT nanoflakes separated by a micrometer‐scale gap. Overall, the proposed room‐temperature unconventional magnetoresistance in vdW FGT‐based heterostructures with the top layer consisting of metallic Pt ensures better compatibility with CMOS integration, opening up new avenues for the design and construction of high‐performance memory devices, deserving of in‐depth future investigations.

## 2 Results and Discussion

**FIGURE 1:**
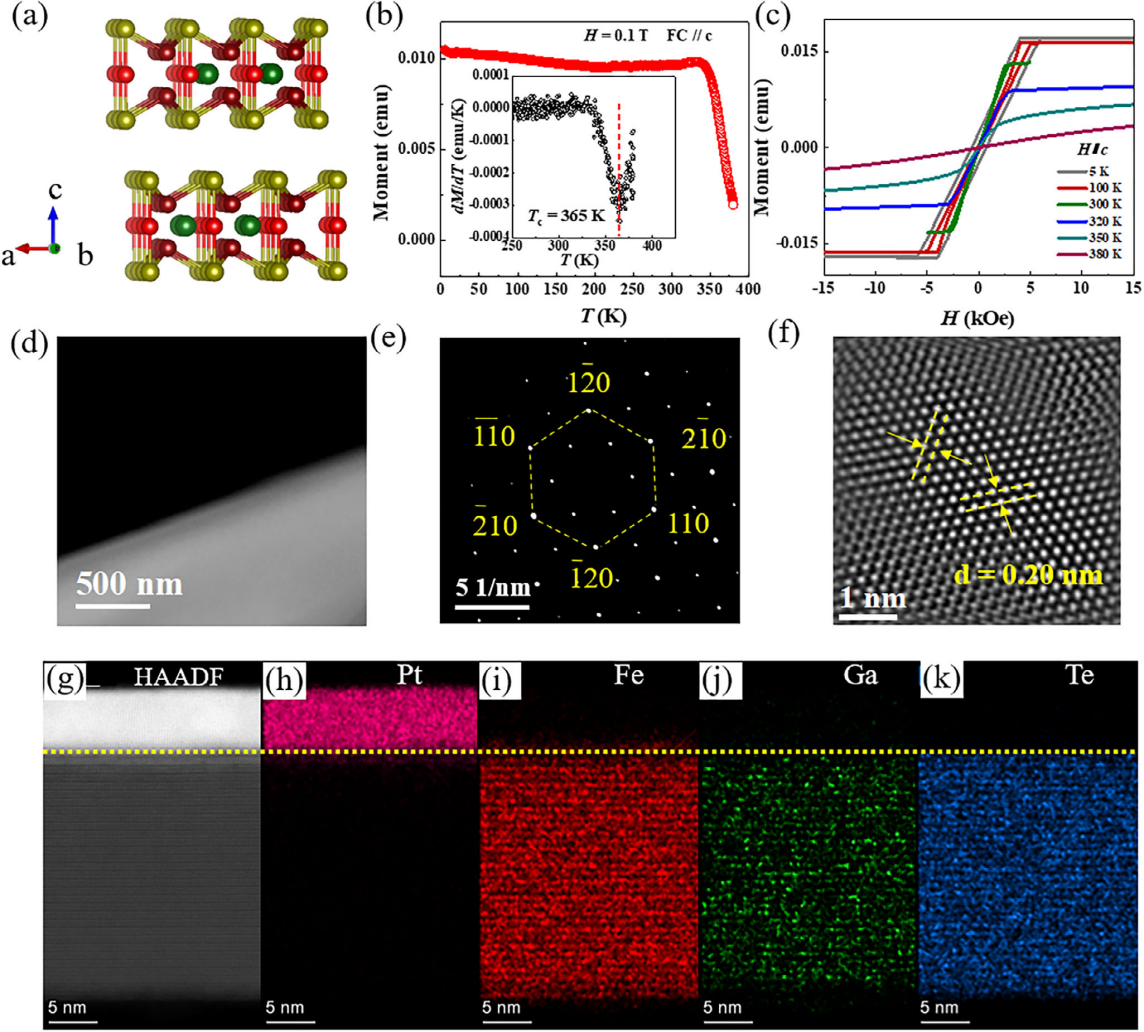
(a) Crystal atomic structure of the FGT. (b) Magnetic moment versus temperature curve of the FGT crystal. The applied magnetic field is 0.1 T. (c) Field‐dependent magnetic moment of the FGT at varying temperatures. (d) TEM image of the FGT. (e) Electron diffraction pattern of the FGT. (f) High‐resolution TEM image of the FGT. (g–k) Cross‐sectional HAADF‐STEM image (g) and corresponding element mappings (h–k) of Pt (pink), Fe (red), Ga (green), and Te (blue) in FGT(23 nm)/Pt(7 nm) heterostructure. The yellow dashed line shows the interface between FGT and Pt layers.

In this work, FGT was first mechanically exfoliated to reduce its thickness, and then directly exfoliated onto oxidized Si substrates to form vdW ferromagnetic FGT nanosheets. Subsequently, a Pt film was deposited through magnetron sputtering, followed by the fabrication of FGT/Pt Hall bar devices for magneto‐electric transport measurements. As shown in Figure 1a, FGT, as a vdW metallic ferromagnet, crystallizes in the P63/mmc space group, consisting of a Fe/FeGa/Fe heterometallic slab sandwiched between two Te layers. A single‐crystal FGT subjected to out‐of‐plane temperature‐dependent moment and hysteresis loops at temperatures from 5 to 380 K (Figure 1b,c) exhibits intrinsic ferromagnetism with *T*_C_ of ∼365 K (inset in Figure 1b). At temperatures below *T*_C_, the FGT bulk crystal exhibits obvious hysteresis, consistent with previous reports [20]. From the transmission electron microscopy (TEM) images, clear diffraction spots can be observed on the FGT crystal (Figure 1d,e), corresponding to crystal planes and indicating a highly ordered crystal with good single‐crystalline quality. In the high‐resolution TEM image (Figure 1f), lattice fringes with a spacing of 0.20 nm are observed for the FGT crystal, corresponding to the lattice d spacings of (1 1 0) and (1 −2 0) planes that match the values from Figure 1e, suggesting high crystal integrity with a few formed defects [21]. To characterize the interfacial information of the FGT/Pt heterostructure, the interfacial quality of a FGT(23 nm)/Pt(7 nm) heterostructure was characterized by cross‐sectional high‐resolution transmission electron microscopy (HRTEM) and scanning transmission electron microscopy (STEM) combined with energy‐dispersive X‐ray spectroscopy (EDS). Figure S1 is the cross‐sectional HRTEM image of the FGT/Pt heterostructure, indicating a polycrystalline structure of the Pt layer and a distinct interface between the FGT and Pt layers. Figure 1g–k show a high‐angle annular dark‐field (HAADF) STEM image together with the corresponding EDS elemental maps of Pt, Fe, Ga, and Te. The interface between the Pt and FGT layers remains flat, clean, and well defined. In addition, the elemental mapping results reveal that Pt, Fe, Ga, and Te are uniformly distributed within their respective regions, with a sharp interface observed between the FGT and Pt layers, exhibiting negligible interdiffusion and high interfacial quality of the heterostructure.

**FIGURE 2:**
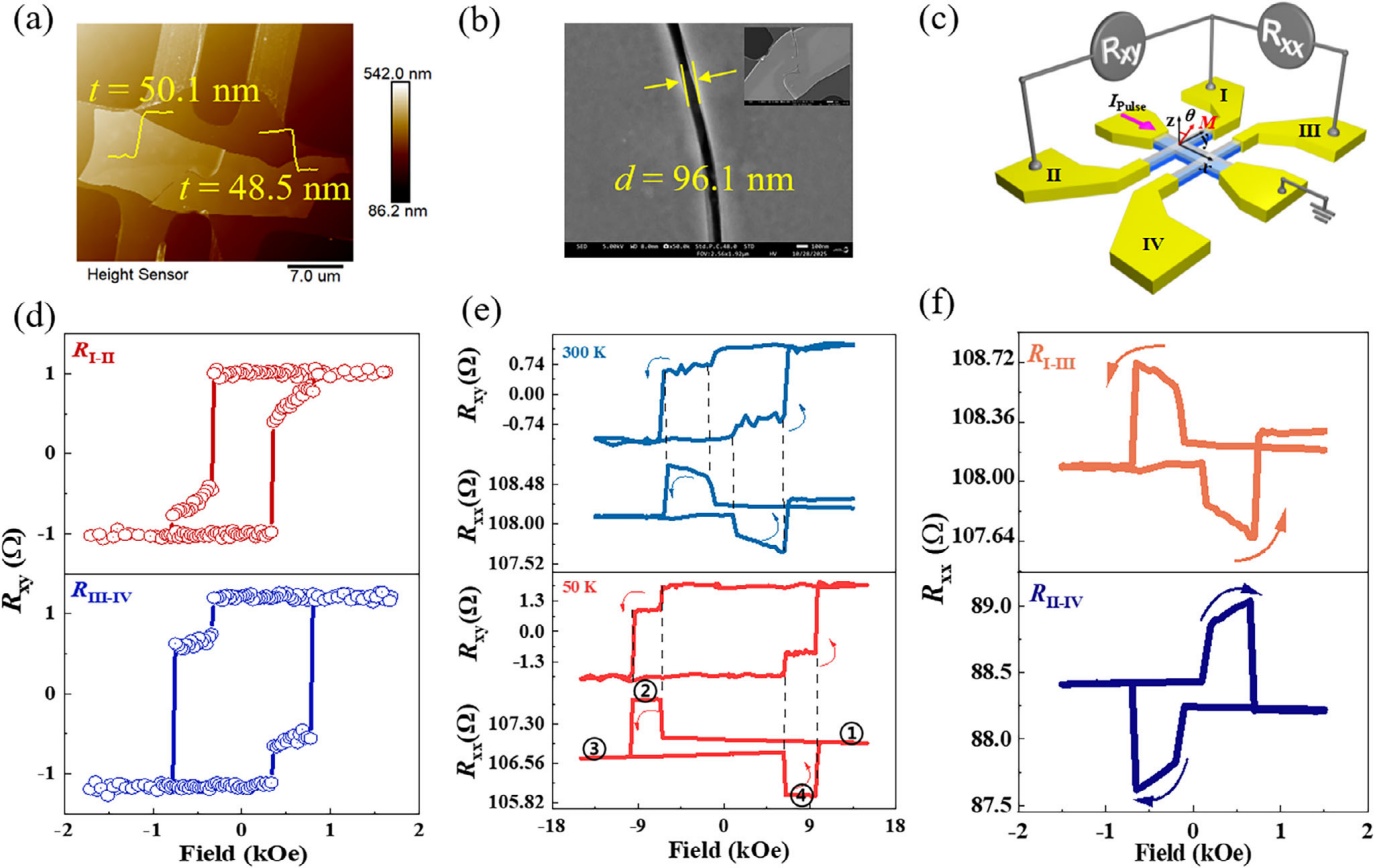
(a) Atomic force microscopy (AFM) image of Device‐1. The thickness of the FGT nanoflakes deposited on the oxidized Si substrate was measured by AFM. (b) SEM image of Device‐1. The gap between the two nanoflakes is ∼96.1 nm. (c) Schematic diagram of the electrical transport measurements. Here, *θ* represents the angle between magnetization (*M*) and the *x*‐axis direction. (d) AHE with different electrodes for Device‐1 measured at room temperature. (e) Correlation between the AHE and AsMR curves measured at room temperature and 50 K. Labels ①, ②, ③, and ④ correspond to Intermediate state I, High‐resistance state, Intermediate state II, and Low‐resistance state, respectively. (f) AsMR effect with different electrodes in Device‐1 at room temperature.

The preparation of thickness‐inhomogeneous FGT nanoflakes via mechanical exfoliation, followed by transfer onto oxidized Si substrates, results in nanoflakes breaking into two pieces along the edge of different thicknesses (Figure 2a), with thickness variation clearly detected by atomic force microscopy (AFM, 48.5 and 50.1 nm for right and left pieces, respectively). Each piece of FGT nanoflake has a uniform thickness. The Pt film with a thickness of ∼8 nm was sputtered onto the sample with a width gap of ∼96.1 nm (Figure 2b), followed by fabrication of a Hall device (Device‐1 in Figure 2a), with the schematic of the electrical transport measurements displayed in Figure 2c. The voltage measurements taken across different contact pairs (I‐II and III‐IV) under applied current suggest the existence of anomalous Hall loops, exhibiting “step‐like” behavior, with a first reversal at ± 60 mT and a second reversal at ± 90 mT. Such “step‐like” behavior in the anomalous Hall curves may arise from the different coercive fields of the FGT and the ferromagnetic Pt interface induced by the proximity effect in Device‐1 (Figure 2d). Further discussion of the “step‐like” behavior is provided later in the AsMR origin section.

Synchronous measurements of Hall resistance (*R*_xy_) and longitudinal resistance (*R*_x__x_) on Device‐1 are illustrated in Figure 2c. At both room temperature and 50 K, *R*_xy_ exhibits a two‐step reversal behavior, while *R*_x__x_ shows an AsMR behavior with four distinct resistance states (Figure 2e). Interestingly, the coercive field of *R*_xy_ coincides with the reversed magnetic fields corresponding to the high‐ and low‐resistance states of *R*_x__x_ (marked with dashed lines in Figure 2e). The same phenomenon is also observed for Device‐2 (Figure S2). Both the coercive field and the reversal field increase with decreasing temperature, as will be discussed in detail below. Since the two FGT nanoflakes were separated in our experiments, the formation of interfacial domain walls can be ruled out, thereby eliminating any contribution from domain wall circulating currents to the AsMR [22, 23, 24]. Furthermore, by swapping the measurement electrodes from I–III to II–IV (Figure 2c), the antisymmetric polarity can also be reversed (Figure 2f). This is consistent with the spin‐momentum locking effect reported previously [9, 14], further confirming that AsMR is not dependent on the interfacial pinning effect [10].

**FIGURE 3:**
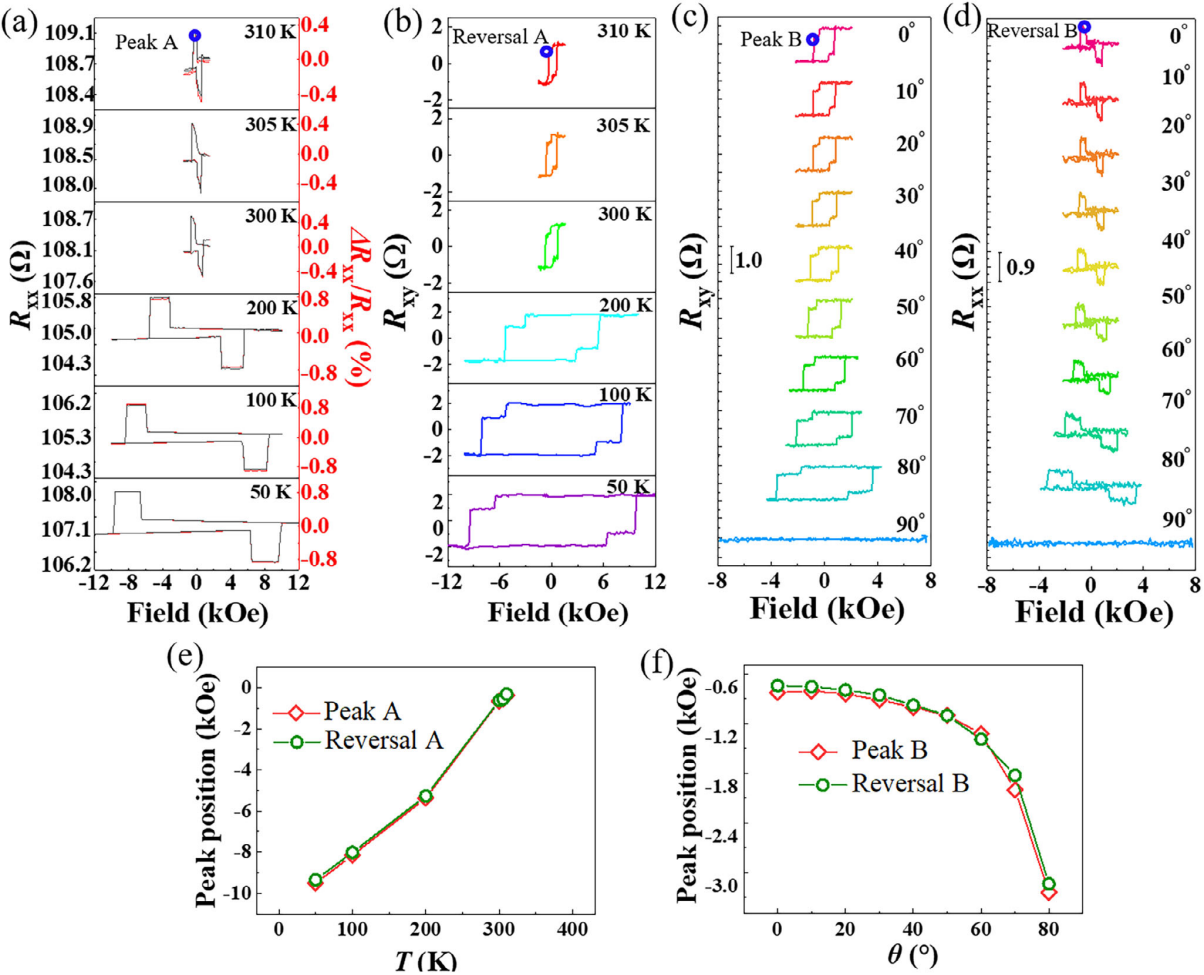
Magneto‐electric transport measurements of Device‐1. (a) AsMR and (b) AHE at different temperatures from 50 to 310 K. (c) AsMR and (d) AHE at different angles under room temperature. The schematic diagram of the measurements is shown in Figure 2c. (e, f) The positions of the antisymmetric peak in *R*_x__x_ and the positions of magnetization reversal in *R*_xy_ as a function of temperature extracted from (a–d).

The magneto‐electric transport measurements of Device‐1 are shown in Figure 3. As displayed in Figure 3a, *R*_xx_ exhibits an AsMR behavior at temperatures from 50 to 310 K. The pronounced enhancement of AsMR at low temperatures is associated with enhanced magnetic ordering, stronger spin‐dependent scattering, and increased interfacial spin polarization [9, 11, 14]. The two‐step reversal behavior of *R*_xy_ can be seen in Figure 3b, corresponding to the asynchronous magnetization reversal of FGT and the ferromagnetic Pt interface. The coercive field of the *R*_xy_ matches exactly the reversed magnetic fields with the high‐ and low‐resistance states, and gradually increases with the decrease in temperature, mainly attributed to the enhanced PMA and suppressed thermal fluctuations [25, 26]. Figure 3c,d shows the dependence of *R*_xx_ and *R*_xy_ on the magnetic field at different angles (*θ*) between magnetization (*M*) and the *x*‐axis direction (0°–90°) at room temperature, respectively. The reversed magnetic fields of *R*_xx_ and the coercive field of *R*_xy_ increase with *θ*. At *θ* = 90°, *R*_xx_ and *R*_xy_ reach zero, due to the completely parallel magnetization of FGT to the *x*‐axis direction. Since the magnetization of FGT is oriented along the out‐of‐plane direction, a stronger magnetic field is required to overcome the strong perpendicular magnetic anisotropy to achieve magnetization reversal [27]. The angle‐magnetoelectric transport data reveal a consistent relationship between AsMR and AHE [28], as illustrated in Figure 3e,f.

**FIGURE 4:**
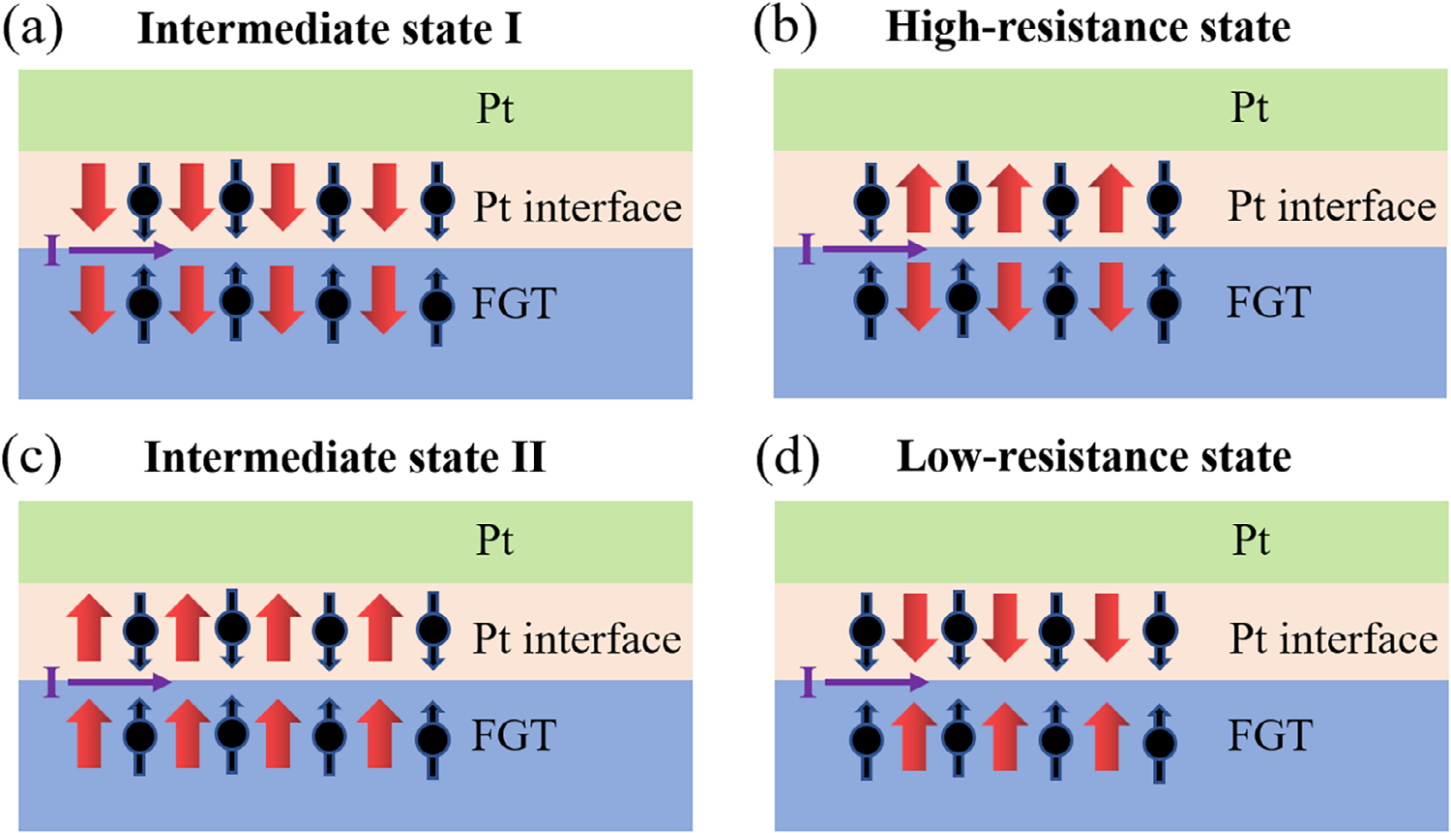
Schematic diagram of the AsMR mechanism for the FGT/Pt devices. The four identified distinct resistance states: intermediate state (a, c), the low‐resistance state (b), and the high‐resistance state (d). Red arrows indicate magnetization in the FGT and interface of FGT/Pt layers, black arrows with spheres denote spin polarization from spin‐momentum locking, and brown arrows represent the charge current.

The AsMR mechanism governing the FGT/Pt devices is illustrated in Figure 4. The magnetization at the Pt interface (the pale red region) adjacent to FGT originates from the magnetic proximity effect induced by the interfacial exchange interaction [29, 30, 31]. Owing to the strong SOC in FGT, spin polarization (black arrows) is generated at the FGT/Pt interface through spin‐momentum locking [9, 14, 32]. Under an applied magnetic field, the spin polarization aligns either parallel or antiparallel to the magnetization direction of the FGT layer and the ferromagnetically polarized Pt interface. In the antiparallel configuration, stronger spin‐dependent electron scattering occurs compared with the parallel configuration, resulting in higher resistance, whereas the parallel configuration corresponds to a lower‐resistance state. Consequently, the resistance state of the device strongly depends on the relative orientation between the interfacial spin polarization and magnetization. The AsMR behavior evolves through four distinct states during the magnetization reversal process, governed by the magnetic configuration and the relative orientation of spin polarization and magnetization in both the Pt interface and FGT layers.

The state where both the interfacial Pt and FGT magnetizations are aligned under a large applied magnetic field is shown in Figure 4a. In this case, the spin polarization is antiparallel to the FGT magnetization and parallel to the interfacial Pt, resulting in an intermediate resistance state (labeled as Intermediate state I in Figure 4a and marked as ① in Figure 2e, according to the resistance relationship in a parallel circuit). In Figure 4b, an antiparallel orientation between the spin polarization and magnetization in both regions, originating from the magnetization reversals in the Pt interface under an applied reversed magnetic field, results in a high resistance state (labeled as High‐resistance state in Figure 4b and marked as ② in Figure 2e). As the reversed magnetic field increases, the spin polarization becomes parallel to the FGT magnetization and antiparallel to the interfacial Pt, giving rise to another intermediate resistance state (labeled as Intermediate state II in Figure 4c and marked as ③ in Figure 2e). In Figure 4d, the low‐resistance state (labeled as Low‐resistance state and marked as ④ in Figure 2e) shows spin polarization and magnetization parallel in the two regions.

To further clarify the relationship between AsMR and the proximity effect‐induced spin polarization in FGT/Pt devices, the FGT/Cr(6 nm) and FGT/Ta(6 nm) heterostructures with completely isolated FGT nanoflakes were fabricated and then patterned into Hall bar devices. The optical images and magneto‐electric transport properties are shown in Figures S3 and S4. It can be seen that the AHE can be observed, while no AsMR can be detected in FGT/Cr(6 nm) Hall bar devices (Figure S3), because the light metal Cr lacks strong SOC. Whereas, both AHE and AsMR are observed in FGT/Ta(6 nm) devices as shown in Figure S4, owing to the strong SOC of Ta. This further verifies that the proximity effect‐induced interfacial spin polarization in Pt plays a key role.

**FIGURE 5:**
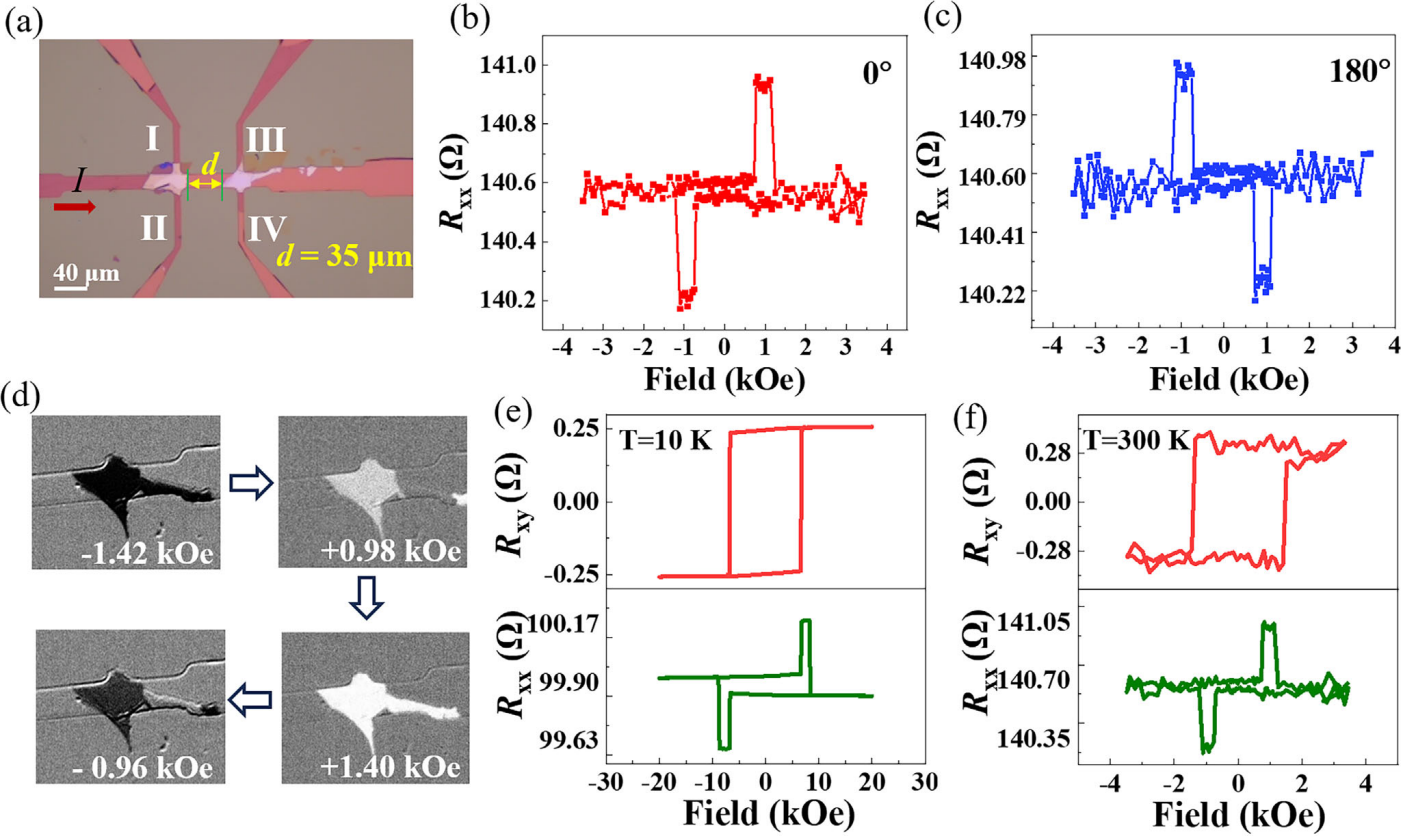
(a) Optical image of FGT/Pt(7 nm) of Device‐3. (b, c) AsMR with the device facing upward (0°) and downward (180°). (d) MOKE images under different applied magnetic fields at 300 K. (e, f) AHE and AsMR in Device‐3 at 300 and 10 K.

To further illustrate the transport mechanism of the AsMR behavior and rule out the possibility that AsMR originates from domain‐wall‐induced circulating currents, an FGT/Pt(7 nm) Hall bar device with a distance of ∼35 µm between the two FGT nanoflakes was fabricated by the same preparation method (labeled as Device‐3). Two FGT nanoflakes of different thicknesses (∼45 nm for the left FGT thickness, ∼35 nm for the right thickness) were positioned at the respective two Hall‐voltage detection sites (Figure 5a), ruling out domain wall formation between the two FGT nanoflakes. The anomalous Hall curves and AsMR measured at different temperatures (Figure S5) are in line with the results of Device‐1. The transverse resistance measurements with the device facing upward and downward were also performed under an applied magnetic field perpendicular to the device surface, and a typical AsMR behavior in both device orientations is displayed in Figure 5b,c. When the device was inverted, the corresponding high‐ and low‐resistance states also reverse. This phenomenon indicates that the AsMR in the FGT/Pt device depends on spin‐momentum locking rather than the interfacial pinning effect [10]. When the device orientation switches between upward and downward, the reversal of high‐resistance and low‐resistance states is attributed to the SOC‐induced Rashba‐split 2D electron gas [33], which gives rise to spin‐momentum locking. In Figure 5d, the magneto‐optic Kerr effect (MOKE) imaging of the right FGT nanoflake under different applied magnetic fields (−1.42 kOe → +0.98 kOe → +1.40 kOe → − 0.96 kOe) illustrates a first reversal of the ferromagnetic Pt interface under a small field, followed by the FGT under a large field. The four magnetic domain states indicate a two‐step reversal of the magnetizations in FGT and the ferromagnetic Pt interface, corresponding to four distinct resistance states in AsMR curves (Figure 5c). These results provide direct evidence of an existing interfacial magnetization due to the proximity effect. The magnetic‐field dependence of *R*_xy_ and *R*_xx_ at room temperature and 10 K (Figure 5e,f) reveals no signal reversal of AsMR at high and low temperatures, further confirming that AsMR does not originate from interface pinning. The same phenomenon is also observed in Device‐4 (∼40 µm between the two FGT nanoflakes) and Device‐5 (∼46 µm between the two FGT nanoflakes), as shown in Figures S6 and S7. Therefore, the AsMR behavior in our experiments primarily originates from the interfacial spin‐momentum locking, ruling out the contributions from the domain wall‐induced circulating currents and interface pinning. Additionally, the current‐dependent measurements of *R*_xy_ and *R*_xx_ for Device‐3 were carried out, as shown in Figure S5c,d. Both the anomalous Hall resistance and the AsMR remain nearly unchanged with increasing current, indicating that the FGT/Pt devices possess good thermal stability.

## 3 Conclusion

In summary, we experimentally confirmed the presence of the pronounced AsMR effect with four resistance states derived from the room‐temperature spin‐momentum locking. Using two completely isolated FGT nanoflakes and measurement approaches with swapping electrodes and flipping device orientations, our findings rule out the contribution to AsMR from domain wall–induced circulating currents and interface pinning. In addition, room‐temperature AsMR behavior was also observed in the Hall bar devices with two FGT nanoflakes separated by a micrometer‐scale gap. Overall, this discovered physical mechanism of AsMR may have important implications for low‐power spintronic devices, deserving further studies and future implementation.

## 4 Experimental Section

### 4.1 Device Fabrication

The bulk Fe_3_GaTe_2_ (FGT) crystals were purchased from Hefei Kejing Co., Ltd. The microstructural characterization of the sample was performed using transmission electron microscopy (TEM, JEM‐F200). The as‐received material was first mechanically exfoliated several times with 3 M blue transparent tape to reduce its thickness, followed by direct exfoliation onto oxidized Si substrates to obtain FGT nanosheets inside a glovebox. The samples were overlaid with a Pt (7 nm) film using a magnetron sputtering system. FGT/Cr and FGT/Ta heterostructures were fabricated using the same method. The base pressure of the sputtering chamber was 4 × 10^−10^ Torr. The Ar pressure was set to 1.7 mTorr for the growth of the Pt and Ta layers, and to 2.5 mTorr for the growth of the Cr layer. The sputtering powers for Pt, Ta, and Cr were 15, 20, and 30 W, respectively. The electrodes for Hall devices were patterned via the laser direct writing technique (MicroWriter ML3, Quantum Design) and etched using an etching machine (K‐150, Advanced Mems). The positive photoresist S1805 was uniformly coated on the surface of the sample through high‐speed spin coating with a speed of 8000 r/min. The exposure dose was set at 60 mJ/cm^2^, and the development time was 60 s. The etching power was 20 W, and the cooling water temperature of the sample stage was 5°C, with an etching duration of 7 min. Subsequently, ultrasonic photoresist removal was performed using acetone to obtain the FGT/Pt heterostructure. The cross‐sectional microstructural characterization of the FGT/Pt heterostructure was characterized by transmission electron microscopy (Thermo Fisher, Talos F200X). The samples were then subjected to optical microscopy (BX51, OLYMPUS) under alternating bright‐field and dark‐field imaging modes, as well as atomic force microscopy (AFM, Bruker Dimension Icon) in tapping mode to determine their local target and precise thickness, respectively. The gap width between FGT nanoflakes in the devices was measured by scanning electron microscopy (SEM, JSM‐IT800). Magneto‐optic Kerr effect (MOKE, Truth Instruments) microscopy was used for direct observation of the magnetic domain reversal.

### 4.2 Magneto‐Electric Transport Measurements

A physical property measurement system (PPMS, Quantum Design Inc.) was utilized for all magneto‐electric transport measurements by selecting the resistivity option. Except for angle‐dependent AHE measurements, a magnetic field perpendicular to each sample was utilized during magneto‐electric transport measurements. The magnetic field‐ and temperature‐dependent *R*_xy_ and *R*_xx_ were determined using standard six‐terminal Hall devices with a constant current of 0.5 µA. The AHE and longitudinal resistance were measured in the temperature range of 50–310 K.

## Author Contributions

**Jinjian Guo**: methodology, funding acquisition, data curation. **Fan Gong**: investigation, data curation, methodology. **Jinnan Liu**: data curation, investigation. **Wei Zhang**: investigation, data curation, writing – review and editing, methodology. **Yunwen Zhu**: writing – original draft, data curation, investigation, methodology. **Xiaolin Luo**: investigation, data curation, methodology. **Baijie Zhu**: investigation, data curation. **Jianlei Shen**: funding acquisition, investigation, data curation. **Zhiyong Quan**: conceptualization, writing – review and editing, funding acquisition, data curation, investigation, project administration, supervision. **Zhuang Liu**: methodology. **Xiaohong Xu**: project administration, writing – review and editing, funding acquisition, conceptualization, supervision.

## Conflicts of Interest

The authors declare no conflicts of interest.

## Supporting information




**Supporting File**: advs76053‐sup‐0001‐SuppMat.docx.

## Data Availability

The data that support the findings of this study are available from the corresponding author upon reasonable request.
