# Supplementary material for: Unconventional Room‐Temperature Antisymmetric Magnetoresistance in van der Waals Fe3GaTe2/Pt Heterostructures
Source: Adv Sci (Weinh). 2026 Jun 9:e76053. Online ahead of print. doi: 10.1002/advs.76053 (PMC13337099; doi:10.1002/advs.76053)
Supplement: Supplementary file 1 — Supporting File: advs76053‐sup‐0001‐SuppMat.docx. [file ADVS-9999-e76053-s001.docx]

Supporting Information

**Unconventional Room-Temperature Antisymmetric Magnetoresistance in van der Waals Fe_3_GaTe_2_/Pt Heterostructures**

***Yunwen Zhu****^1,2^, Xiaolin Luo^2^, Fan Gong^2^, Jinnan Liu**^1,2^, Zhuang Liu^1,2^, Jianlei Shen^1,2^, Jinjian Guo^3^, Baijie Zhu**^1,2^, Wei Zhang^1,2,3,*^ Zhiyong Quan**^1,2,3,^**^*^* ***and Xiaohong Xu****^1,2,*^*

^1^School of Materials Science and Engineering, Key Laboratory of Magnetic Molecules and Magnetic Information Materials of Ministry of Education, Shanxi Normal University, Taiyuan 030031, China

^2^Research Institute of Materials Science, Shanxi Key Laboratory of Advanced Magnetic Materials and Devices, Shanxi Normal University, Taiyuan 030031, China

^3^Instrumental Analysis Center, Shanxi Normal University, Taiyuan 030031, China

Corresponding authors. E-mails: Wei Zhang (zhangwei_aic@sxnu.edu.cn), Zhiyong Quan (quanzy@sxnu.edu.cn), Xiaohong Xu (xuxh@sxnu.edu.cn)

**1. Cross-sectional HRTEM image of FGT/Pt heterostructure**


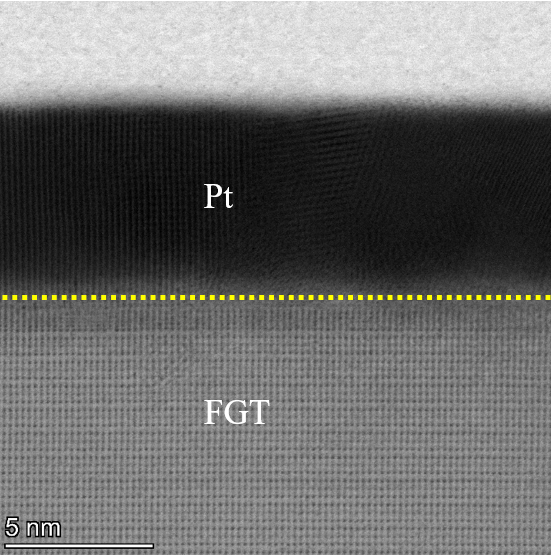


**Figure S1.** Cross-sectional HRTEM image of FGT(23 nm)/Pt(7 nm) heterostructure. The interface between FGT and Pt layers is indicated by the yellow dashed line.

**2.** **Magneto-electric transport properties of Device-2**


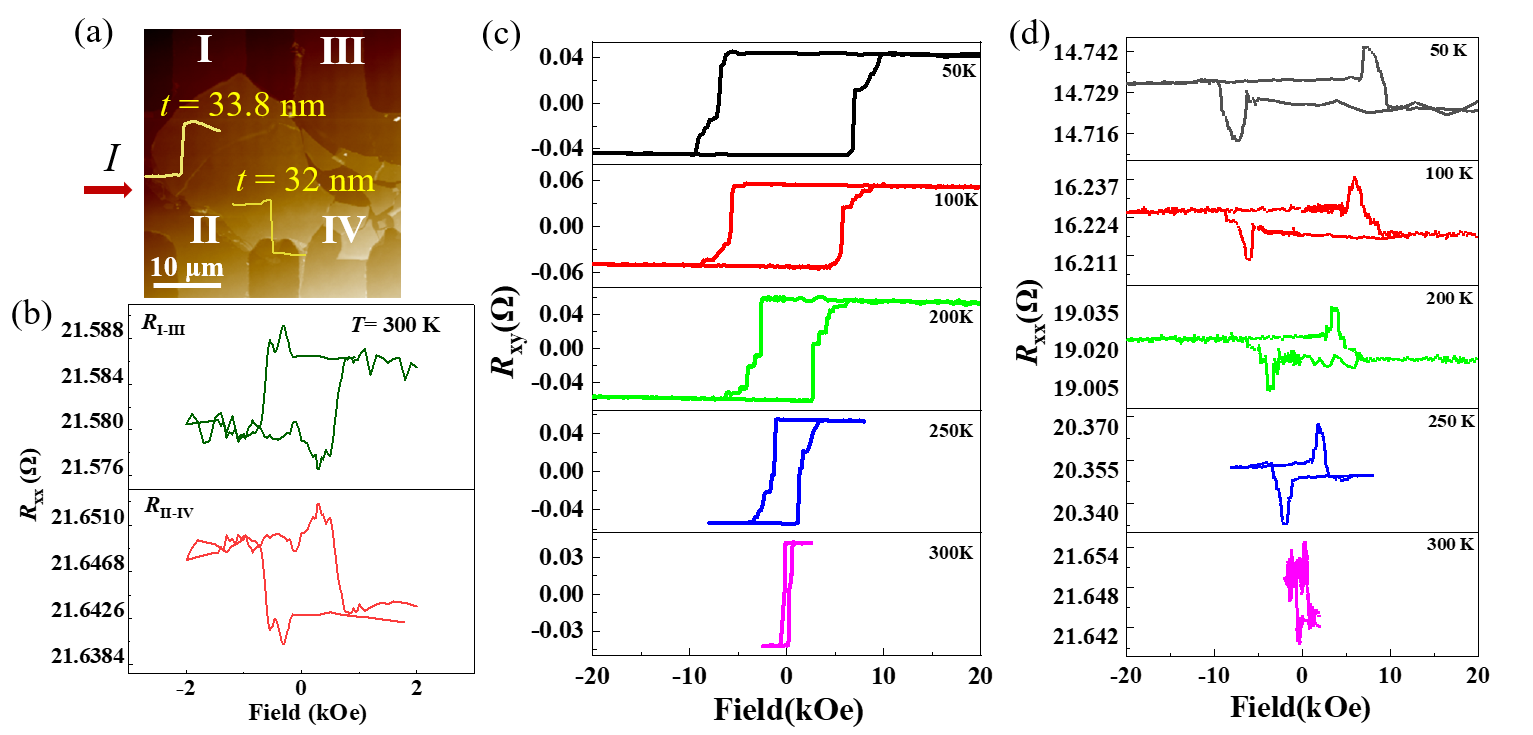


**Figure S2.** (a) AFM image of Device-2 consisting of FGT/Pt(7 nm) with cracks. (b) AsMR with different electrodes measured at room temperature. By swapping the measurement electrodes from I-III to II-IV, the antisymmetric polarity also reverses. (c,d) AHE and AsMR at different temperatures.

**3. Magneto-electric transport properties of FGT/Cr devices**


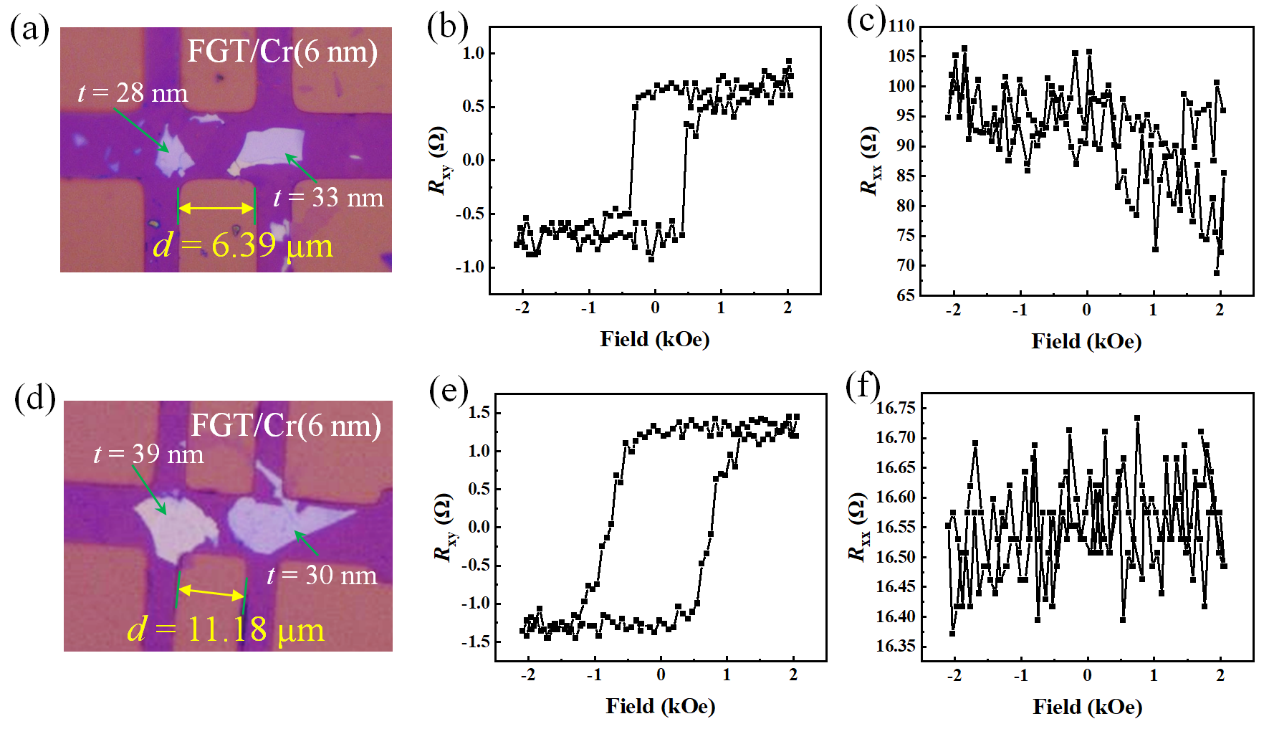


**Figure S3.** (a,d) Optical images of FGT/Cr(6 nm) Hall bar devices. AHE (b,e) and AsMR (c,f) measured at room temperature.

**4. Magneto-electric transport properties of FGT/Ta device**


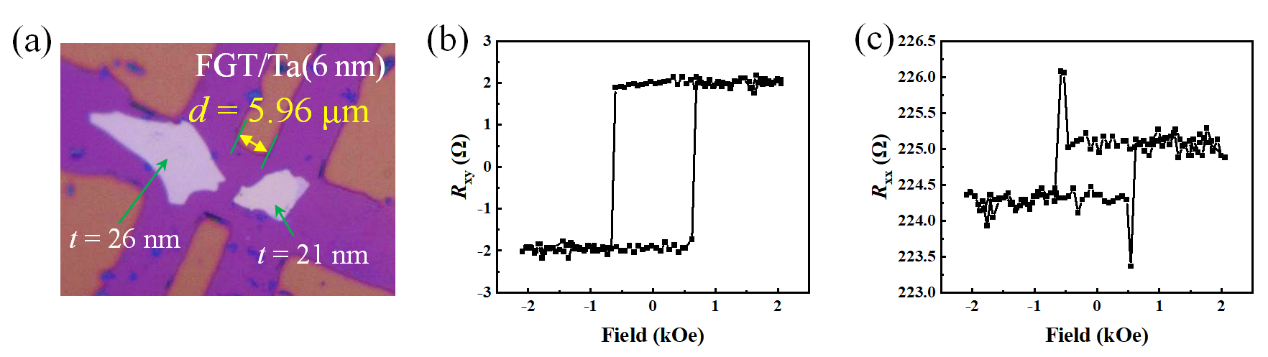


**Figure S4.** (a) Optical image of FGT/Ta (6 nm) Hall bar device. AHE (b) and AsMR(c) measured at room temperature.

**5. The AHE and AsMR effect in Device-3**


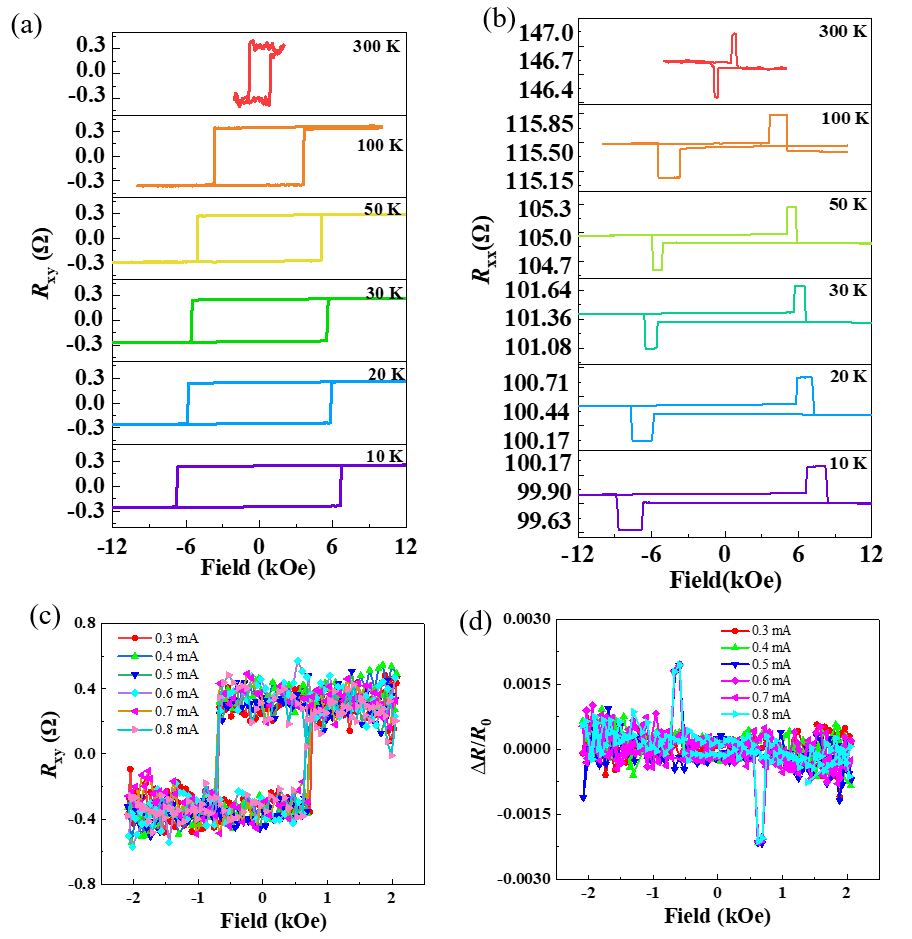


**Figure S5.** (a) AHE and (b) AsMR measured at different temperatures. The coercive field of *R*_xy_ coincides with the reversed magnetic fields corresponding to the high- and low-resistance states of *R*_xx_. (c) AHE and (d) AsMR measured under various currents.

**6. The optical image, thickness, and AsMR in Device-4**

**
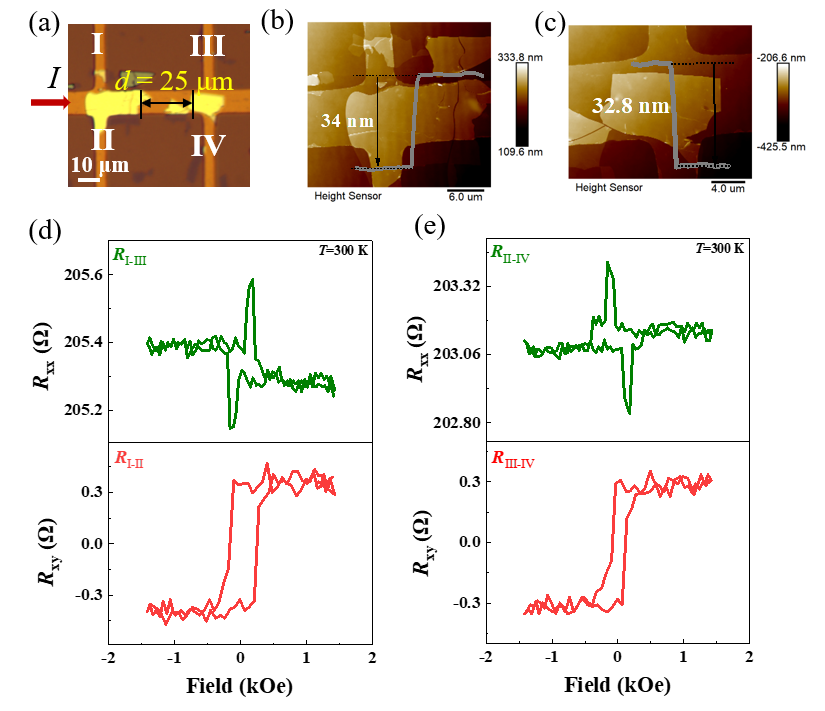
**

**Figure S6.** (a) Optical image of Device-4 composing of FGT/Pt(7 nm) with a gap of ~25 μm between the two FGT nanoflakes. (b,c) The thickness of the FGT nanoflakes measured by AFM, with the left nanoflake ~34 nm (b) and the right ~32.8 nm(c). (d,e) AsMR and AHE with different electrodes measured at room temperature.

**7. The optical image, thickness, and AsMR in Device-5**

**
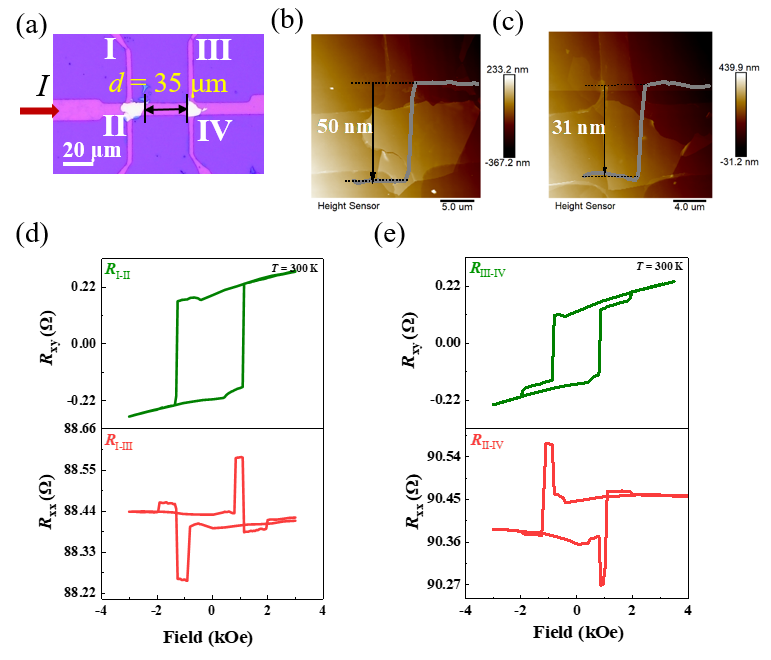
**

**Figure S7.** (a) Optical image of FGT/Pt(7 nm) in Device-5, (~35 μm between the two FGT nanoflakes). (b,c) The thickness of the FGT nanoflakes in Device-5 measured by AFM, with the left (b) nanoflake being ~50 nm and the right (c) one ~31 nm. (d,e) AHE and AsMR with different electrodes in Device-5 at room temperature. The tiny resistance plateaus and step-like signals in AsMR may originate from the influence of micro-nano fabrication processes of Device-5.
